# Research and clinical translation of trilayer stent-graft of expanded polytetrafluoroethylene for interventional treatment of aortic dissection

**DOI:** 10.1093/rb/rbac049

**Published:** 2022-07-22

**Authors:** Gang Wang, Caiyun Gao, Benhao Xiao, Jie Zhang, Xunyuan Jiang, Qunsong Wang, Jingzhen Guo, Deyuan Zhang, Jianxiong Liu, Yuehui Xie, Chang Shu, Jiandong Ding

**Affiliations:** State Key Laboratory of Molecular Engineering of Polymers, Department of Macromolecular Science, Fudan University, Shanghai 200438, China; R&D Center, Lifetech Scientific (Shenzhen) Co., Ltd., Shenzhen 518057, China; State Key Laboratory of Molecular Engineering of Polymers, Department of Macromolecular Science, Fudan University, Shanghai 200438, China; R&D Center, Lifetech Scientific (Shenzhen) Co., Ltd., Shenzhen 518057, China; R&D Center, Lifetech Scientific (Shenzhen) Co., Ltd., Shenzhen 518057, China; State Key Laboratory of Molecular Engineering of Polymers, Department of Macromolecular Science, Fudan University, Shanghai 200438, China; State Key Laboratory of Molecular Engineering of Polymers, Department of Macromolecular Science, Fudan University, Shanghai 200438, China; State Key Laboratory of Molecular Engineering of Polymers, Department of Macromolecular Science, Fudan University, Shanghai 200438, China; R&D Center, Lifetech Scientific (Shenzhen) Co., Ltd., Shenzhen 518057, China; R&D Center, Lifetech Scientific (Shenzhen) Co., Ltd., Shenzhen 518057, China; R&D Center, Lifetech Scientific (Shenzhen) Co., Ltd., Shenzhen 518057, China; Department of Vascular Surgery, the Second Xiangya Hospital of Central South University, Changsha 410011, China; State Key Laboratory of Cardiovascular Diseases, Center of Vascular Surgery, Fuwai Hospital, National Center for Cardiovascular Diseases, Chinese Academy of Medical Science and Peking Union Medical College, Beijing 100037, China; State Key Laboratory of Molecular Engineering of Polymers, Department of Macromolecular Science, Fudan University, Shanghai 200438, China

**Keywords:** aortic dissection, stent-graft, expanded polytetrafluoroethylene, delivery system for interventional treatment, clinical translation of biomaterials

## Abstract

The aortic dissection (AD) is a life-threatening disease. The transcatheter endovascular aortic repair (EVAR) affords a minimally invasive technique to save the lives of these critical patients, and an appropriate stent-graft gets to be the key medical device during an EVAR procedure. Herein, we report a trilayer stent-graft and corresponding delivery system used for the treatment of the AD disease. The stent-graft is made of nitinol stents with an asymmetric Z-wave design and two expanded polytetrafluoroethylene (ePTFE) membranes. Each of the inner and outer surfaces of the stent-graft was covered by an ePTFE membrane, and the two membranes were then sintered together. The biological studies of the sintered ePTFE membranes indicated that the stent-graft had excellent cytocompatibility and hemocompatibility *in vitro*. Both the stent-graft and the delivery system exhibited satisfactory mechanical properties and operability. The safety and efficacy of this stent-graft and the corresponding delivery system were demonstrated *in vivo*. In nine canine experiments, the blood vessels of the animals implanted with the stent-grafts were of good patency, and there were no thrombus and obvious stenosis by angiography after implantation for 6 months. Furthermore, all of the nine clinical cases experienced successful implantation using the stent-graft and its postrelease delivery system, and the 1-year follow-ups indicated the preliminary safety and efficacy of the trilayer stent-graft with an asymmetric Z-wave design for interventional treatment.

## Graphical Abstract

**Figure:**
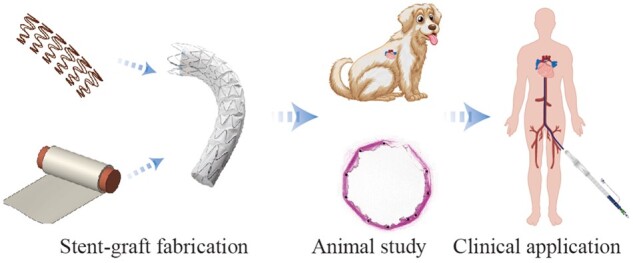

## Introduction

**Figure 1.**
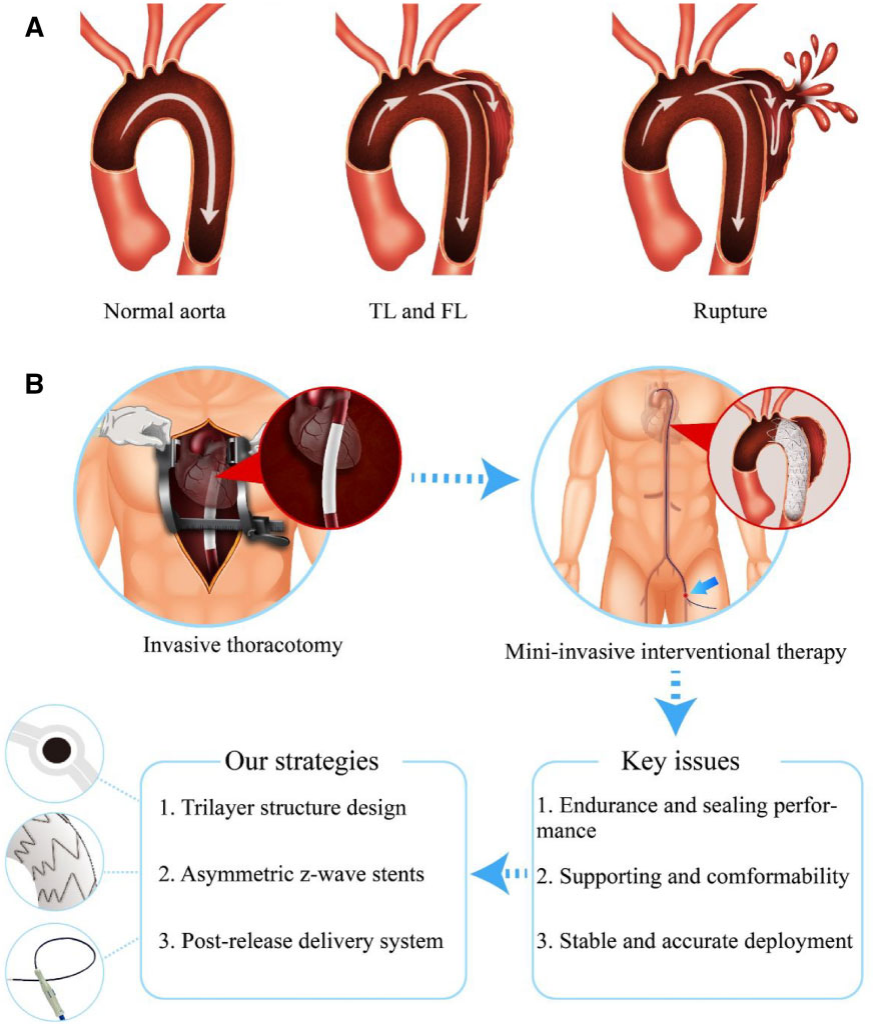
Schematic illustration of the AD formation and treatment. (**A**) Illustration of the process of AD formation. (**B**) The comparison of invasive thoracotomy and minimally invasive interventional treatment using an ePTFE stent-graft. The arrow in the interventional surgery illustration indicates the entry point of the delivery system. Key issues of the interventional device and corresponding design strategies are described in the bottom.

The aortic dissection (AD) is a serious disease that can occur in any position along the aorta. Unlike an aortic aneurysm, AD is caused by some lesions in the intima of the aorta, and common risk factors include hypertension and atherosclerosis [1]. The intima of the aorta is torn under the impact of blood flow firstly, and then the intima is separated from the middle and outer layers of the blood vessel wall along the aorta, eventually leading to the formation of true lumen (TL) and false lumen (FL). If left untreated, the FL may become larger and larger with the impact of blood flow pressure and may rupture ultimately. Such a rupture as schematically shown in Fig. 1A will make the patient lose blood quickly, leading to very high mortality.

The Stanford classification is the most widely used method for the evaluation of AD disease [2]. Stanford A dissection (Type A) means that the AD involves the aortic arch or ascending aorta, and Stanford B (Type B) dissection indicates that the AD only involves the descending aorta. Thoracotomy is the preferred treatment method for Type A dissection, which usually involves with the aortic branches such as the brachiocephalic artery, the left common carotid artery or the left subclavian artery (LSA), and thus too complex for endovascular repair [3–6]. For Type B dissection, more and more doctors prefer to perform the endovascular repair with a stent-graft owing to its merits such as higher safety and fewer complications [7–10].

The conventional treatment for aortic aneurysms and ADs is surgery. Doctors need to open the patient’s chest and cease the heart beating prior to establish a cardiopulmonary bypass with an extracorporeal circulation machine. The surgeons need to find the target lesion vessel and remove it and then sew the ends of the artificial graft around the cut sites to replace the diseased aorta. This procedure has high risk and is time-consuming, and even a successful operation is frequently associated with serious complications such as heart, brain and renal insufficiency [11–13]. The surgical treatment is especially unsuitable for elderly people.

In 1991, Parodi *et al.* [14] first reported the implantation of an intraluminal graft to isolate the abdominal aortic aneurysms by using retrograde cannulation of the common femoral artery. With the rapid development of this technique, more and more physicians tried to cure aortic aneurysms and dissections by using modified devices. In 1994, Dake *et al.* [15] reported 13 cases of stent-graft implantation by using transluminal method to treat aneurysms and dissections in descending thoracic artery. In the medical field, the interventional method to treat aortic aneurysms is called endovascular aortic repair (EVAR), and thoracic EVAR is called TEVAR. In the past 20 years, this kind of medical technique has been developed rapidly with the modification of the stent and stent-graft [16–18]. The stent-graft system usually consists of membrane and stent. The membrane keeps the blood away from the dissection FL, and thus decreases the blood pressure in the FL and avoids the rupture of the aorta. A satisfactory stent-graft may endure more than 10 years in the human body without performance compromised. A practical stent-graft system for interventional treatment contains at least a membrane, a stent skeleton, and a delivery system. The endurance and sealing performance of the selected membrane is very important to the stent-graft [19]. The stent skeleton provides the supporting force to fix the stent-graft in the aorta and affects the stent-graft conformability to the tortuous aorta. The delivery system as a device is used to deliver and release the stent-graft in a proper position in the human body, and therefore, the stability and accurate deployment performance are important for such advanced medical device.

Since the first implantation of stent-graft into the human descending thoracic aorta, doctors and engineers have been trying to modify the stent-graft structure to better adapt to aortic lesions anatomy of the patients [14, 15, 20–22]. With the development of the implantation materials and delivery technology [23–26], the stent-graft for interventional operation has been developing rapidly [27–30]. Some manufacturers have produced the commercial stent-graft for clinical use. The most common mode of the thoracic stent-graft is a tubular graft constructed by metallic stents and a polyester fabric graft, such as Talent^™^ and Valiant^™^ stent-graft (Medtronic vascular), Zenith^™^ TX and TX2 stent-graft (Cook Medical Inc.) [31–34]. The fabric graft is sewn onto the stents with sutures to prevent unintentional movement between them. The polyester fabric graft can keep the blood flow away from the aortic aneurysm or AD, while the metallic stents expand the graft and attach it to the vessel wall. Gore TAG^™^ is another thoracic aortic stent-graft, which is composed of an expanded polytetrafluoroethylene (ePTFE) membrane and supporting nitinol stents [35]. The stents are fixed on the ePTFE graft by some fluorinated ethylene propylene strips separately, and these stents can move from each other to ensure the flexibility of the stent-graft to adapt to the aorta anatomy.

The interventional procedure of a stent-graft is schematically presented in Supplementary Fig. S1. All the existing products of stent-graft in the market have their limitations. It is still difficult to find a stent-graft system with excellent comprehensive performance to treat AD via interventional treatment. To tackle this problem, this study will focus on solving some of these limitations.

Different from the common Dacron stent-graft in which a poly(ethylene terephthalate) graft is sewed on to the stents with sutures, we aim to design an ePTFE stent-graft with a trilayer structure, in which the metal stent is sandwiched between two ePTFE membranes. This special structure is expected to prevent blood leakage from the sewing holes. Moreover, from the stent design standpoint, we designed an asymmetric Z-wave stent, which has long and short Z-waves on the greater curvature side and lesser curvature side of the stent-graft, respectively. This design as schematically presented in Fig. 1B might endow the stent-graft with the capability of excellent conformability to adapt with the curved aorta.

As a necessary and supplementary medical accessory for a stent-graft in interventional treatment, the delivery system enables loading a stent-graft in a sheath, delivering it to the target lesion and deploying it at the accurate setting position. In this study, we designed a ‘post-release’ delivery system, which can fix the proximal end of the stent-graft in the right position during stent-graft deployment. Displacement of stent-graft may occur in a challenging procedure due to the flush of the blood and complex anatomy; in contrast, we can, taking advantage of the ‘post-release’ delivery system, place the stent-graft more accurately.

Key issues of the interventional device and corresponding design strategies are presented in the lower part of Fig. 1B. In order to evaluate the *in vivo* properties of this new type ePTFE stent-graft, nine animal studies were carried out in canine models. Supplementary Table S1 shows the evaluation methods of a stent-graft and its delivery system and the evaluation criteria in animal studies and clinical trials. The success rate of the technique in our animal study was 100%, and all animals were followed up for 6 months. The results of anatomy data confirmed that the blood vessels of all the animals were of patency, and there was no thrombus and obvious stenosis. Furthermore, we assessed the safety and efficacy of this ePTFE stent-graft in human trials to treat patients with AD, and the clinical studies further confirmed the feasibility of our medical device.

## Materials and methods

### Fabrication of the ePTFE stent-grafts for interventional treatment

The proposed ePTFE stent-graft consists of two ePTFE membranes and one nitinol skeleton. The nitinol skeleton is made of nitinol wire with a diameter of 0.3–0.5 mm, in which the mass content of nickel is 54.5–57%, and the rest are titanium and trace elements. The nitinol skeleton or stent was manufactured by connecting a series of Z-shape wave rings in a welding process. The ePTFE membrane was covered on a cylinder SUS304 mold bar for 5–6 layers, then the stents were capped onto the inner ePTFE membrane surface and the outer ePTFE membrane was also covered with 5–6 layers. Finally, this assembled device was placed in an oven and underwent a heat treatment process to make the ePTFE membrane sintering. After that, the trilayer stent-graft was formed. The stent-graft detached from the mold bar and the excessive ePTFE membranes at both ends of the stent-graft were trimmed neatly.

### Physicochemical characterizations of sintered ePTFE membranes

We made the characterizations of the polymer membranes from several aspects. The sintered ePTFE membrane was prepared by the method described in the supporting information.

The membrane morphology was observed with a field-emission scanning electron microscope (Ultra55, Zeiss, Germany). The specimens were sputter-coated with gold under 10 mA for 90s and scanning electron microscopy (SEM) images were taken at accelerating voltage of 2 kV.

The tensile testing of the ePTFE membrane was carried out with an SANS CMT4104 tester. The specimens were prepared by cutting an ePTFE membrane into strips of 15 mm × 200 mm along the direction of axial direction and circumferential direction, respectively. The ePTFE membrane was stretched at a speed of 200 mm/min on the testing machine until the strips broke. The stress-strain curves were analyzed following the conventional way [36].

The crystalline phase structure of ePTFE was detected with X-ray diffraction (XRD, Bruker D8 diffractometer, Germany) in the range of 10–80°. The ePTFE membrane was cut into a size of 2 × 2 cm and laid flat in the sample slot of the device for testing.

Thermal properties of the ePTFE membrane were detected with differential scanning calorimetry (DSC, TA Q2000, USA). The thermal behavior of ePTFE membrane was analyzed by the DSC curve in an operated temperature range from 40°C to 400°C at a heating rate of 10°C/min.

Surface wettability was also examined. Water contact angle (WCA) was tested with an optical device (JC2000DM, Zhongchen). Briefly, 10 µl of deionized water was dropped on the membrane surface, then an image was taken with a video camera. The WCA was determined on the screen of the monitor using the SCA 20 software.

### *In vitro* biocompatibility of sintered ePTFE membranes

The cytotoxicity evaluation is based on a cell counting kit-8 (CCK8) assay as previously reported [37]. In brief, viability of human umbilical vein endothelial cells was detected by CCK8 assay in culture media after adding the indicated liquid. The ePTFE membrane was cut into small round flakes with an average diameter of nearly 15 mm and kept in culture media at 37°C for 24 h to obtain the extracted liquid. The culture media and 0.1 mg/ml sodium dodecyl sulfate were set as the blank and positive controls of cytotoxicity, respectively. For each group, *n *=* *5. The characterization follows the standard of acceptable cytotoxicity according to ISO 10993-5:2009.

We also carried out the *in vitro* hemolysis assay. The test method is as follows. Took 8 ml of fresh anticoagulant rabbit blood and diluted it with 10 ml of 0.9% NaCl solution. The diameter of the ePTFE membrane was 3 cm. Upon addition of 5 ml of normal saline, the system was incubated at 37°C for 30 min, and the contact area between the sample and the physiological saline was strictly in accordance with ISO 10993-12:2012. After that, 0.1 ml of diluted anticoagulant blood was added and the incubation continued for 60 min at 37°C. Next, we carried out centrifugation at 800 g for 5 min, and the supernatant was taken to measure the absorbance at 540 nm with a multifunction microplate detector (BioTek, Cytation3, USA). Distilled water and normal saline were used as positive and negative controls, respectively (*n* = 5 for each group). The characterization follows the standard of acceptable hemolysis according to ISO 10993-4:2002.

### Characterization methods of the stent-graft and the delivery system

In order to detect radial force of a stent-graft, two semicircular concave blocks were assembled onto the electronic mechanics tester, and then the stent-graft was fixed between the two blocks. Then, we compressed the stent-graft from the diameter direction by 20%, and the maximal value of compression force is defined as the radial force of the stent-graft.

We specifically examined the release force and postrelease force in order to check the feasibility of our delivery system for interventional treatment. Firstly, we loaded the stent-graft in the delivery system and assembled the handle tension meter with the grip or the postrelease button of the delivery system separately. Then, the grip or postrelease button was pulled back to release the stent-graft or release the proximal end of the stent-graft. The maximal force values during the above-mentioned two processes are defined as release force and postrelease force.

### Animal studies of the stent-graft system

All protocols were approved by the Lifetech Institutional Animal Care and Use Committee. Following ISO 25539-2:2012 Cardiovascular implants Endovascular device-Part 2: Vascular stents and 3R Principle (Reduction, Replacement, Refinement), nine adult dogs were included into the animal study of the stent-graft. After a conventional anesthesia and disinfection, the delivery system loaded with stent-graft was introduced to the animal aorta through the femoral artery under the guidance of digital subtraction angiography (DSA, Wandong Medical, China). When the proximal end of the stent-graft was positioned near the second branch artery of the aortic arch, the stent-graft was deployed by operating the handle of the delivery system. After implantation, the puncture sites were closed and the animals were allowed to recover.

All experimental animals received postoperative intramuscular injections of ceftiofur sodium (3–5 mg/kg) for 3 days. The animals were kept for 6 months and received food and water three times daily. Oral aspirin (100 mg) was administered for 30 consecutive days.

The dietary habits, health, mental status and bowel activities of the animals were observed after procedure and during a 6-month follow-up. At the end of the study point, all animals were euthanized and dissected. After the aorta with implantation device was extracted from the animal body, it was fixed with formalin and then imbedded in methyl methacrylate. Afterwards, then the specimens were sliced and stained with hematoxylin and eosin (HE).

### Clinical studies of the stent-graft system

After the approval of the hospital ethics committee, nine patients with Type B ADs were recruited in the Second Xiangya Hospital of Central South University, following the informed consents of the patients and good clinical practice guidelines. Before operation, each patient under computed tomography angiography (CTA) examination to verify that the patient was suitable for stent-graft implantation. The stent-grafts were implanted into the patients under the standard interventional operation procedure. Each patient underwent CTA examination at 1, 6 and 12 months after the procedure to evaluate the ADs and stent-graft performance.

## Results

### Structural characteristics of the trilayer ePTFE stent-graft

**Figure 2.**
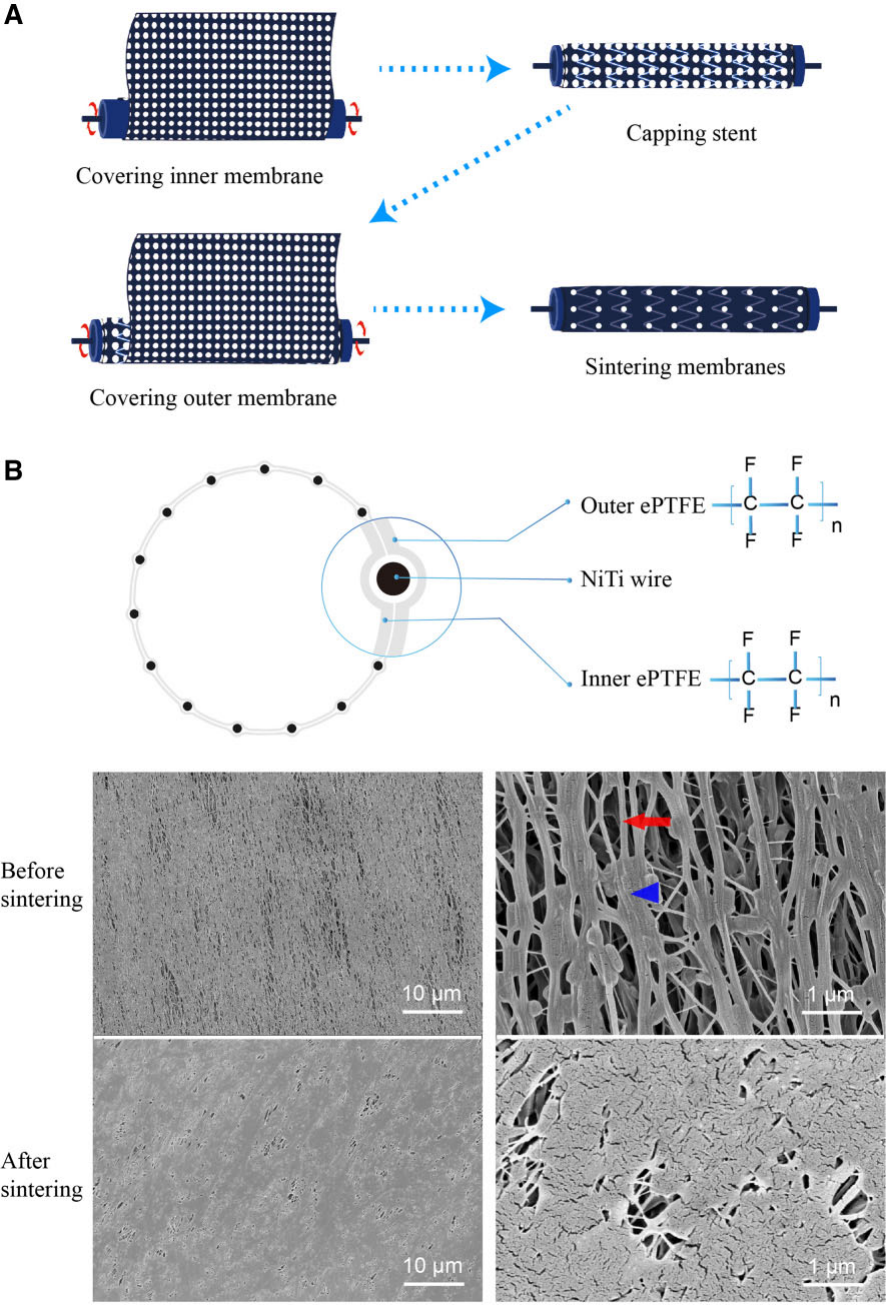
Schematic presentation of fabrication of the ePTFE stent-graft with a trilayer structure. (**A**) The fabrication process of the ePTFE stent-graft. (**B**) Trilayer structure illustration of the stent-graft and SEM images of the ePTFE membrane before and after sintering. The ePTFE membrane before sintering exhibits node-fiber structure. As demonstration, a node is shown by the triangle, and a fiber is shown by the arrow.

The trilayer ePTFE stent-graft was manufactured by a ‘composite sintering’ process as shown in Fig. 2A. The acquired stent-graft was flexible because of the porous structure of the ePTFE membrane. The inner ePTFE membrane and outer ePTFE membrane were eventually bonded together with the nitinol stent frame between them (Fig. 2B).

The raw material of ePTFE membranes is composed of vertically and horizontally interwoven fibers and nodes (Fig. 2B). This special ‘node-fiber’ structure endows the ePTFE membrane with good flexibility and other special properties, such as waterproof and air permeability [38]. The SEM images showed that the fibers in the ePTFE membrane were obviously oriented along the circumferential direction, which was induced by the different degrees of longitudinal and transverse stretchings of the patented biaxial stretching process. The circumferential direction and the axial direction of the stent-graft are shown in Supplementary Fig. S2. After sintering, a significant decrease of the pores among the fibers was observed, and obvious fusion occurred among the fibers. The sintering of the ePTFE membranes significantly improved the membrane strength of the stent-graft.

### Physical and biological characteristics of the sintered ePTFE membranes of the stent-graft

**Figure 3.**
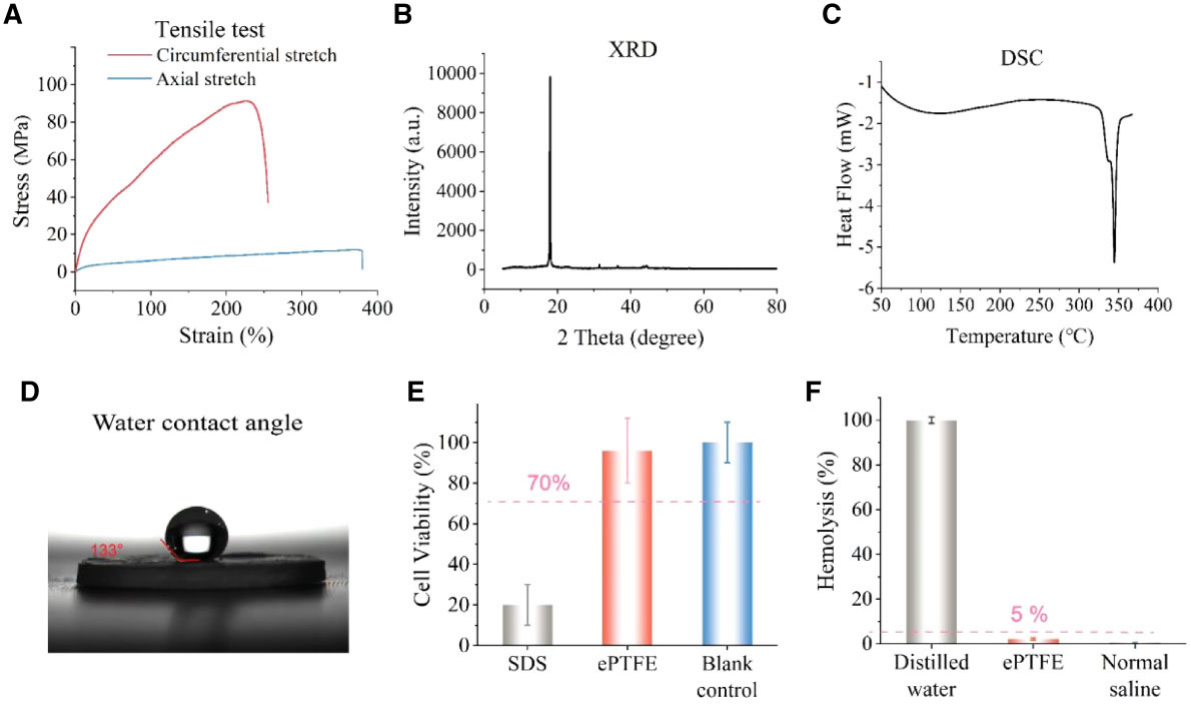
Physical and biological characteristics of the as-prepared ePTFE membrane of the stent-graft. (**A**) Tensile tests of sintered ePTFE membrane. (**B**) XRD pattern of the ePTFE membrane. (**C**) DSC curve of the ePTFE membrane, indicating that its melting point is 342°C. (**D**) Hydrophilicity test of the ePTFE membrane. (**E**) Cytotoxicity test of the ePTFE membrane. (**F**) Hemolysis test of the ePTFE membrane. The dashed lines in (E) and (F) indicate the criteria of the corresponding ISO standards to access *in vitro* biocompatibility of a medical device.

Physical and biological properties of the sintered ePTFE membranes of the stent-graft were characterized. Figure 3A shows the tensile stresses of the ePTFE membrane along the axial and circumferential directions. The resultant circumferential stress of the ePTFE membrane was about seven times of the axial stress. The higher circumferential stress provides the membrane sufficient strength in the circumferential direction to resist the blood pressure when the stent-graft is implanted in the aorta.

The crystalline phase of ePTFE was characterized with XRD. As shown in Fig. 3B, a sharp peak appeared at 18° in the XRD curve, reminiscent of a (100) reflection at 2θ = 18° of crystalline PTFE [39]. Meanwhile, we examined the differential scanning calorimetry (DSC) curve (Fig. 3C). The results showed that the melting point of the ePTFE was 342°C and the melting enthalpy (Δ*H*_m_) of the ePTFE was 53.2 J/g. Based on the Δ*H*_m_ of pure PTFE 71.0 J/g [40], the ePTFE membrane exhibited a crystallinity of 74.9% as calculated by the ratio of the two Δ*H*_m_ values. The results are consistent with the previous reports that the Δ*H*_m_ of PTFE decreased with the manufacture process, such as sintering, melt shrinkage and fiber formation [41].

As shown in Fig. 3D, the ePTFE membrane with a WCA of 133° was hydrophobic, consistent with the report about ePTFE [38]. According to the tests of cytotoxicity (Fig. 3E) and hemolysis (Fig. 3F), the experimental groups exhibited a statistically significant difference with the positive control, and no statistical difference with the blank or negative control. The cell viability of ePTFE membrane read 96%, significantly better than the acceptance standard of 70%. The hemolysis of the ePTFE read 2.2%, lower than the standard of an accepted hemolysis of 5%. The results of cytotoxicity and hemolysis demonstrated that our sintered ePTFE membrane had excellent biocompatibility and hemocompatibility *in vitro*.

### Fabrication and characterization of the stent-graft

**Figure 4.**
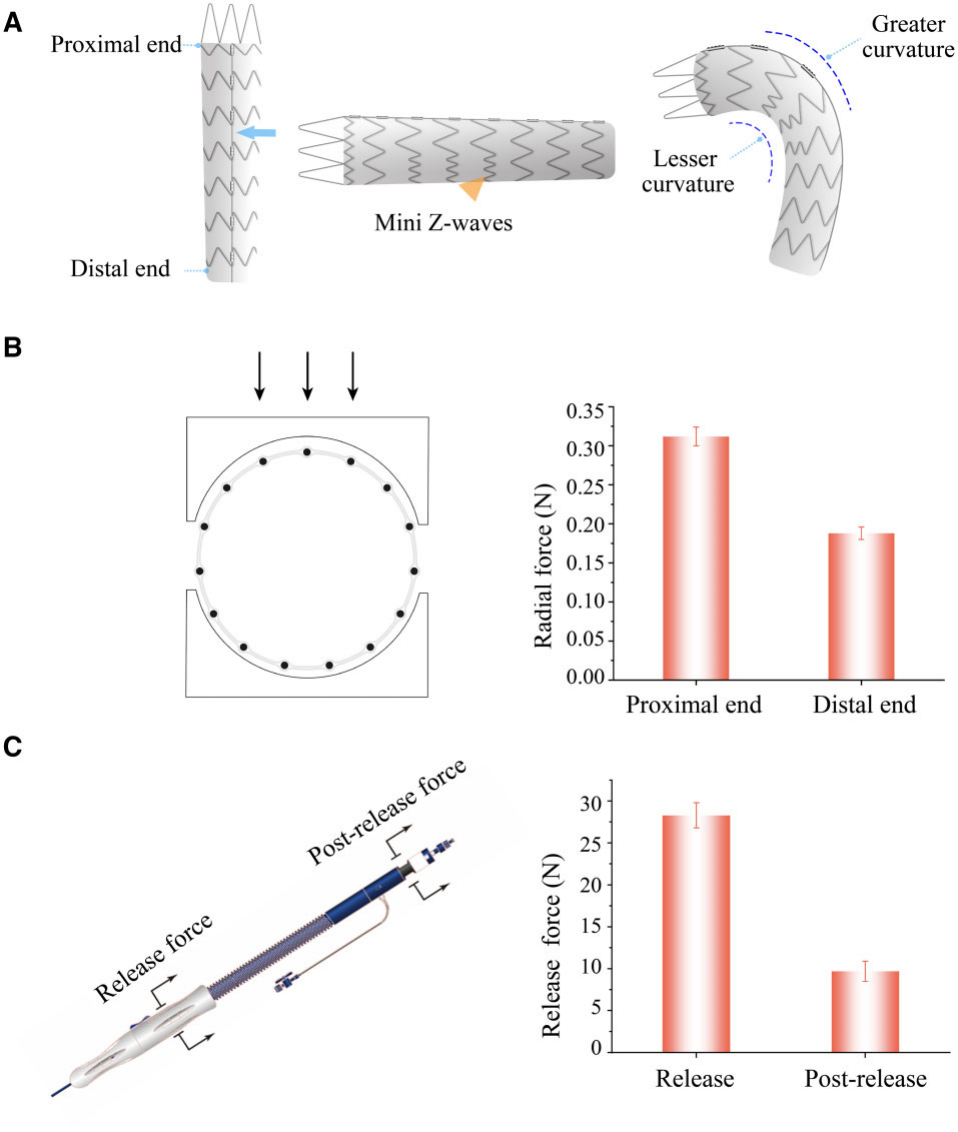
Schematic diagram of the stent-graft and characterization of the stent-graft and delivery system. (**A**) Schematic diagram of the stent-graft and the superiorities of the stent-graft design. The connecting bar on the back of the stent-graft (as the arrow shown) provides the stent-graft with good supporting performance in the axial direction, and the mini Z-wave stents (as the triangle shown) on the lesser curvature side of the stent-graft provide the stent-graft with excellent compatibility to the aortic arch. (**B**) The measured radial forces of the stent-graft. (**C**) The release force and postrelease force of the delivery system when deploying the stent-graft. The measured release force of the stent-graft is <30 N and the measured postrelease force is <10 N, which indicate the feasibility for doctors to operate the delivery system.

The stent-graft was designed with an asymmetric Z-wave nitinol stent. The mini Z-waves are, as indicated by the triangle in Fig. 4A, shorter on the lesser curvature side of the descending aorta than on the other side. Such an asymmetry design endows the stent-graft with excellent compatibility to the curved anatomy of the aorta. There exists a connecting bar on the back of the stent-graft (as the arrow shown) to connect all the Z-wave rings, and this novel design provides the stent-graft sufficient axial strength when it is deployed in the vessel. The connecting bar can also prevent shortening of the stent-graft axially.

We also determined the radial forces of the proximal end and the distal end of the stent-graft, which read about 0.32 and 0.18 N in Fig. 4B, respectively. The adequate radial force of the stent-graft provides the stent-graft sufficient anchoring force in the blood vessel and thus avoids the stent-graft displacement under the impact of the blood flow. The radial force of the proximal end of the stent-graft is larger than that of the distal end in order to provide a better anchoring force against the aorta wall to fix the stent-graft in the position. The distal end force is a little weak, which is beneficial for reducing the stimulation to the vessel wall to prevent intima tear from happening again at the distal end of the stent-graft.

We particularly measured the release force and postrelease force during a stent-graft deployment with a delivery system using our designed test methods. As shown in Fig. 4C, the release force and the postrelease force of our delivery system were <30 and 10 N, respectively, indicating a good operability for a physician.

### Fabrication and characterization of the delivery system

**Figure 5.**
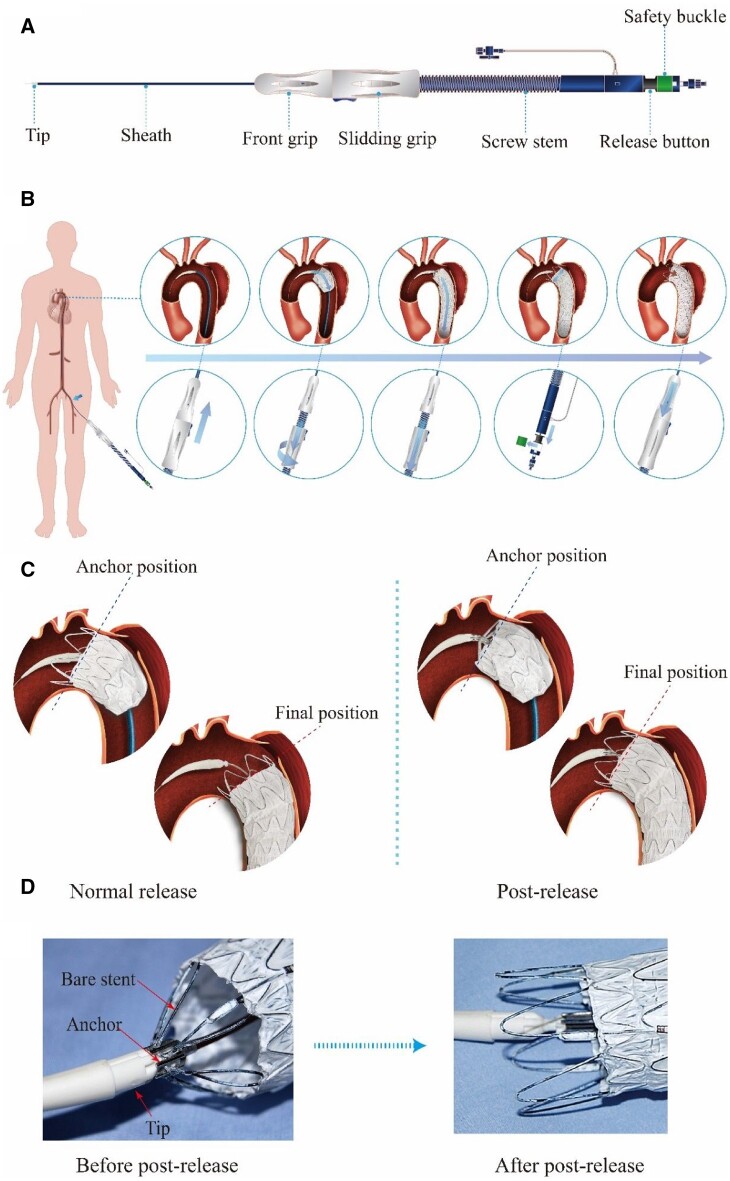
The postrelease delivery system and the implantation procedure. (**A**) Illustration of the delivery system. (**B**) Diagram of the stent-graft delivery process, including the enlarged view of the process steps of the stent-graft in the aorta and the flow chart of the operation of the delivery system outside the human body. (**C**) Schematic illustration of the comparison of the way of normal release and postrelease. In the normal release mode, the anchor position of the stent-graft may be changed due to the flush of the blood; and in the postrelease mode, the anchor position is firmly fixed by the anchor of the delivery system. (**D**) Illustration of the postrelease delivery system captured with and without stent-graft. The left picture demonstrates that the bare stent was captured by the anchor of the delivery system and the proximal end of the stent-graft cannot move during the stent-graft deployment; and the right picture demonstrates that the bare stent was released by withdrawing the anchor of the delivery system.

The delivery system mainly consists of a sheath tube and an integrated handle (Fig. 5A). The stent-graft is compressed and loaded in the proximal part of the sheath tube. The handle contains several parts, which are used for operating the deployment of the stent-graft.

The operational procedures to implant a stent-graft in human body with our postrelease delivery system are illustrated in Fig. 5B. First, delivered the sheath from the femoral artery until the proximal part of the sheath reached the target lesion of the aorta. Second, under the guidance of DSA, aligned the proximal end of the stent-graft with the anchor point of the aorta, then rotated the sliding grip to pull the sheath back to deploy the proximal part of the stent-graft. Third, pulled the sliding grip back directly to deploy the rest of the stent-graft more quickly. Fourth, removed the safety buckle and pulled the release button back to release the proximal capture mechanism (‘post-release’ mode) of the stent-graft. Finally, withdrew the delivery system and left the stent-graft in the aorta to exclude the blood from the broken aortic wall. A typical demonstration video of deploying a stent-graft with a delivery system is provided in the Supplementary Video S1.

The delivery system with postrelease mode is more accurate than the normal release mode of delivery system when deploying the stent-graft, as shown in Fig. 5C. In the normal release mode, the anchor position of the stent-graft may be changed due to the flush of the blood; however, in the postrelease mode, the anchor position is firmly fixed by the anchor of the delivery system. Figure 5D shows the capture state and the release state of the proximal end of the stent-graft. The left demonstrates that the bare stent was captured by the anchor of the delivery system and the proximal end of the stent-graft could not move during the stent-graft deployment; and the right demonstrates that the bare stent was released by withdrawing the anchor of the delivery system. When deploying the proximal part of the stent-graft, the postrelease system can capture the proximal bare stent to avoid the displacement of the stent-graft under the impact of blood flow.

### Animal studies in a canine model

**Figure 6.**
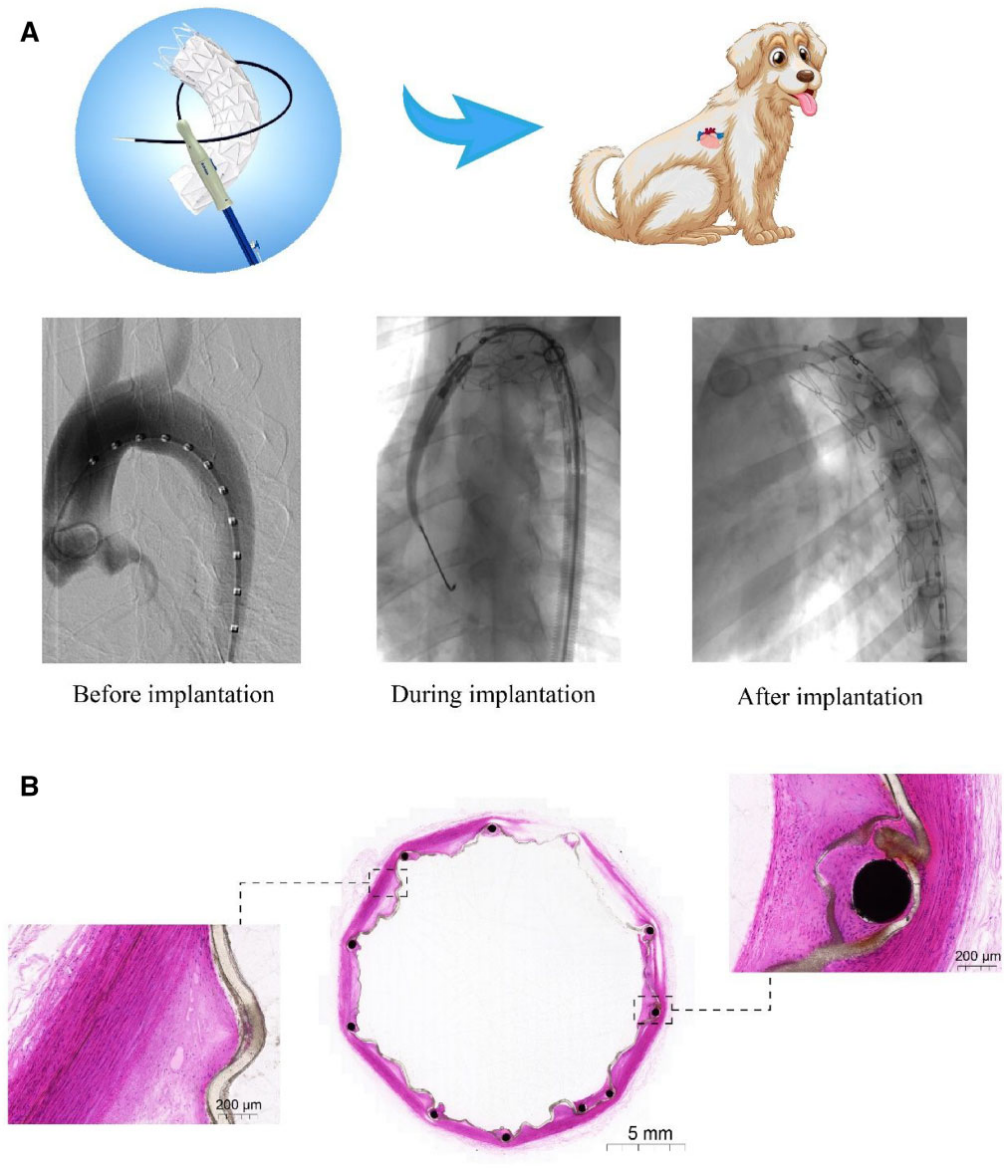
Evaluation of the stent-graft in an interventional treatment in a canine model. (**A**) DSA images of an experimental canine model, including before, during and after stent-graft implantation. (**B**) HE staining images of implantation sites. The middle is a whole view of the cross-section in the stent-graft in the animal artery, which indicates good supporting performance of the stent and no thrombus in the lumen. The right is a local view at the site of Ni–Ti wire embraced by the ePTFE membrane, indicating the in-growth of smooth muscle cells and fibroblasts inside and outside the membrane. The left is a local view of the membrane and the vessel wall, showing a good fit between the membrane and the hyperplastic intima of the vessel. The hyperplastic tissue shows neovascularization and no inflammatory cell infiltration. The stent-graft was implanted in the animal’s aorta for 6 months.

The performance of the novel-designed stent-graft and delivery system were evaluated in a canine model. As shown in Fig. 6A, the procedures were checked or guided under the DSA guidance, including the preimplantation, during and after the stent-graft implantation. Nine stent-grafts were successfully implanted into the desired and appropriate position of the nine canines using the methods described above.

During the 6-month follow-up, all animals were alive and in good health condition. After 6 months, angiography was performed on all animals, and the DSA images indicated that the vessels were of patency and there was neither deformation nor fracture of the stents.

All the animals were sacrificed at the end of the 6-month follow-up. The aorta with stent-graft was removed from each studied animal and fixed for histopathological inspections. The gross inspection of other important organs was also inspected. No abnormalities were found in the lungs, liver, spleen, kidneys or heart.

Figure 6B shows the HE staining images of a stent-graft in the aorta taken from one studied animal. The local view of the membrane and the vessel wall shows a good fit between the membrane and the hyperplastic intima of the vessel. The whole view of the cross-section in the middle of the stent-graft in the animal artery indicates good supporting performance of the stent-graft and no thrombus in the lumen. The local view at the site of Ni–Ti wire embraced by the ePTFE membrane indicates the in-growth of smooth muscle cells and fibroblasts inside and outside the membrane. Furthermore, the hyperplastic tissue shows neovascularization and no inflammatory cell infiltration. Therefore, the materials of the stent-graft have good biocompatibility.

We summarized the comprehensive performances of the delivery system and stent-graft in the animal experiments in Table 1. The evaluation methods and criteria of our delivery system and stent-graft are listed in Supplementary Table S1. The statistical results illustrated that the stent-graft implantation was successful in our animal studies.

**Table 1.** Evaluation of the delivery system and stent-graft performance in nine canine models

Performance indexes	Evaluation result-grade, *n* (%)				
	I	II	III	IV	V
Positioning accuracy of delivery system				1 (11)	8 (89)
Operational controllability of delivery system				2 (22)	7 (78)
Stability of delivery system				1 (11)	8 (89)
Supporting performance of stent-graft				1 (11)	8 (89)
Conformability of stent-graft			1 (11)	2 (22)	6 (67)
Attachment ability of stent-graft				2 (22)	7 (78)
Visibility of stent-graft marker				4 (44)	5 (56)

We also examined thrombus formation on the sintered ePTFE membranes in canine models. Preparation of the ePTFE membranes and the methods of animal implantation as described in the Supplementary material. Some typical results are shown in Supplementary Fig. S3. The left is the global view of a vessel sample and an ePTFE specimen implanted in a dog jugular vein for 4 h, and the right is an SEM image of a sintered ePTFE membrane specimen taking from the animal’s body. According to the protocol recommended by ISO 10993-4:2017: Biological evaluation of medical devices–Part 4: Selection of tests for interactions with blood, the thrombus was minimal on the ePTFE membrane.

### Clinical applications

**Figure 7.**
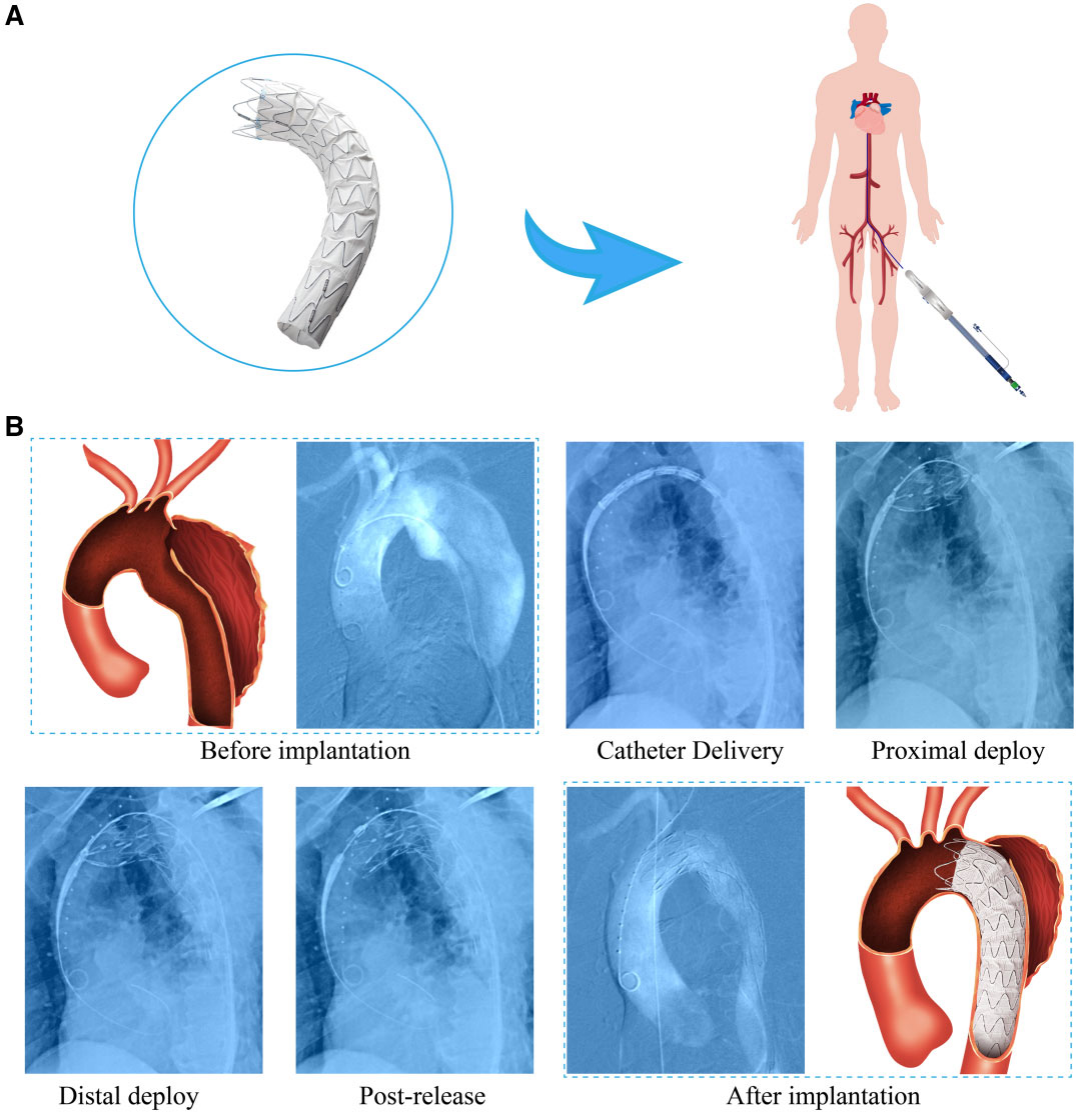
Global view of an ePTFE stent-graft and a demonstration of a human clinical case of a 66-year-old man. (**A**) Global view of an ePTFE stent-graft and schematic diagram of human intervention method. (**B**) DSA images of a human clinical case during various stages of the procedure.

We applied this new stent-graft system in nine clinical cases with Stanford B ADs. In particular, a typical case is shown in Fig. 7. This is a 66-year-old man with a thoracic intimal tear at the site of 2 cm away from the LSA branch. The angiography of preimplantation shows that the FL is very big and the TL is very small due to the compression of the FL. If left untreated, the FL may grow faster under the impact of the blood flow, which would eventually lead to rupture and life-threatening. Thus, we suggested this patient to do a TEVAR by using this new stent-graft system, which was approved by the committee and agreed by the patient.

During the procedure, the sheath loaded with the stent-graft was delivered from the femoral artery to the target lesion in the thoracic aorta. Under the guidance of DSA, the proximal end of the stent-graft was aligned with the position near the opening of LSA branch. Then the stent-graft was deployed and the proximal end of the stent-graft was released by operating the handle. The final angiography shows that the stent-graft was well-positioned, and the FL was not visible as the intimal tear entry was occluded by the stent-graft. The detailed DSA images of the procedure for this patient are provided in the Supplementary Video S2.

**Figure 8.**
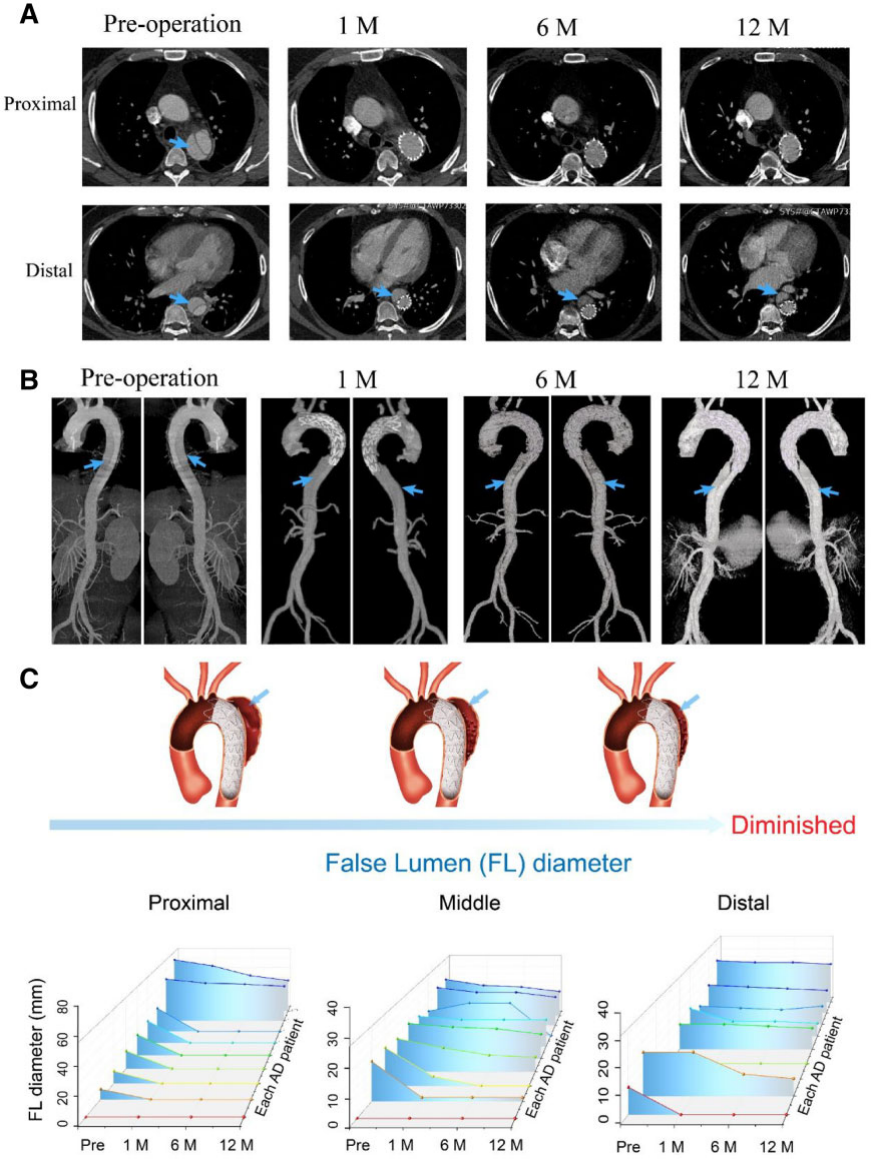
Human clinical case and follow-up evaluation. (**A**) Cross-section images of CTA after stent-graft implantation in an enrolled patient, including preoperation, 1 , 6 months and 1-year postoperation. (**B**) Three-dimensional reconstruction of CTA images of the same person, including preoperation, 1, 6 months and 1-year postoperation. (**C**) Statistical data analysis of the diameter changes of vascular FL after stent-graft implantation of nine patients with type B ADs, including the proximal, middle and distal FL diameters.

We also record the follow-ups. Figure 8A shows the CTA images of one of the enrolled patients, including the CTA image of preoperation and 1, 6 and 12 months of follow-up after the operation. The aorta was separated into TL and FL (as indicated by the arrows) at the proximal part and distal part preoperation. After the procedure, the FL at the proximal part disappeared at the following-up stage; however, the FL still existed at the distal part and its diameter decreased with the increasing of follow-up period.

The CTA images for pre- and after implantation of stent-graft in the same person were reconstructed, with some results shown in Fig. 8B. The AD originated from the aortic arch near to the LSA branch from the preoperation 3D image. In addition, the interfaces between the TL and FL were identified from the 3D images, as indicated by the arrows. After stent-graft implantation, the FL almost disappeared at the stent-graft implanted position; that is because the stent-graft occluded the proximal intima tear and expanded the TL, resulting in the shrinkage or disappearance of the FL. As reported in the literature [42, 43], at the distal of the aorta without stent-graft, the FL still existed because of no stent-graft supporting the TL, and thus FL was still filled with blood from other distal intima tear or other branch vessels.

We did an analysis of the data of the FL diameters preoperation and postoperation of the nine patients. The data of each patient are summarized in Fig. 8C, and the statistical results of diameter changes of vascular FL before and after stent-graft implantation in nine patients with type B ADs are listed in Supplementary Table S2. The result shows that the diameter of the FL decreased dramatically after the stent-graft implantation, especially at the proximal part of the stent-graft implantation.

The statistical results of the comprehensive performance evaluation of the delivery system and stent-graft in the nine human clinical cases are shown in Table 2. One case of positioning accuracy and operational controllability of delivery system gets Grade IV, and all other performance indexes get the highest Grade V. Hence, our new design of the stent-graft and delivery system has great advantages.

**Table 2.** Delivery system and stent-graft performance evaluation in nine human clinical cases

Performance indexes	Evaluation result-grade, *n* (%)				
	I	II	III	IV	V
Positioning accuracy of delivery system				1 (11)	8 (89)
Operational controllability of delivery system				1 (11)	8 (89)
Stability of delivery system					9 (100)
Supporting performance of stent-graft					9 (100)
Conformability of stent-graft					9 (100)
Attachment ability of stent-graft					9 (100)
Visibility of stent-graft marker					9 (100)

The follow-up imaging evaluation results are summarized in Table 3. The FL sealing rate and partial or complete thrombosis after operation 1, 6 and 12 months were 100%; and the incidence of endoleak, rupture or significant enlargement of the FL was zero at the 12-month follow-up point. These clinical data supported the success of the trilayer stent-graft.

**Table 3.** Follow-up imaging evaluation up to 12 months after stent-graft implantation in nine patients with type B ADs

Evaluation indexes (postoperation)	Evaluation results (%)
FL sealing rate	100
Endoleak rate	0
Partial or complete thrombosis of FL after 1 month	100
Partial or complete thrombosis of FL after 6 months	100
Partial or complete thrombosis of FL after 12 months	100
Rupture or significant enlargement of FL after 12 months	0

## Discussion

The latest decade has witnessed rapid progress in biomaterials and medical devices, in both fundamental research [44–51] and applications [52–59]. While medical materials have been much developed [60–68], it is still challenging to find a biomaterial-related cardiovascular device appropriate for interventional treatment. Although stent-grafts have been designed and improved a lot, and are actually in clinic use, there are still some problems such as endoleak, stent-graft failure, displacement and so on [69–71]. Herein, we provide a new stent-graft and delivery system. The novel trilayer design of stent-graft endows the stent-graft with good durability because the nitinol stents are capsulated and fixed by the sintered ePTFE membranes (Fig. 2). The trilayer device can protect the nickel–titanium materials from the complex environment of the human body. Furthermore, the integrated ePTFE layer helps to disperse the pressure from the vessel wall, which endows the stent-graft with excellent fatigue resistance performance. The sintered ePTFE membranes are sandwiched around the stent without any suture, so the resultant graft is free of suture holes, which can reduce the incidence of Type IV endoleak [72, 73].

A good stent-graft should have enough flexibility to adapt to the curved aorta anatomy, such as aortic arch, to reduce the straightening force on the proximal end and distal end during the stent-graft bending [31]. This ability to bend and stick to the vessel wall is usually called ‘conformability’ [74]. To this end, we developed an asymmetric Z-wave design for the stent-graft, and thus the stent waves on the lesser curvature (near the small curve side of the aortic arch) are shorter and the counterparts on the greater curvature side are longer (as shown in Fig. 4A). Therefore, the space between the metal waves on the lesser curvature is larger than that on the greater curvature, which provides more space for the mini-waves to move from each other easily. The character endows the stent-graft with good flexibility. And this asymmetric Z-wave design combined with the sintered ePTFE membranes provides a proper softness of the graft, resulting in an excellent conformability. Stent-graft implantation accuracy is highly dependent on the controllability of the delivery system. The delivery system with postrelease mechanism can make sure the implantation of the stent-graft in an accurate position (Fig. 5C).

The sintered ePTFE membrane had sufficient strength to resist the blood pressure when the stent-graft was implanted in the human body (Supplementary Fig. S3). It is worth noting that the circumferential strength of the stent-graft membrane was about seven times that of the axial strength (Supplementary Fig. S2), which was beneficial for the stent-graft to resist the expansion force caused by the blood pressure. The cytotoxicity assay and hemolysis assay indicate the ePTFE membrane has excellent biocompatibility, hemocompatibility and safety *in vitro* (Fig. 3).

The radial force of the proximal part of the stent-graft was larger than that of the distal part, and the non-uniform radial force design better meets the requirements of clinical needs. The proximal part of the stent-graft with a slight larger radial force is responsible for anchoring with the vessel wall and sealing the AD, while the distal part of the stent-graft with a slight less radical force is mainly responsible for being compliant with the aorta and relieving the stimulation to the vessel wall to avoid stent-graft-induced new entry at the distal part of the aorta [75–77]. This special design is especially suitable for the patients in China, where AD with a higher morbidity than aortic aneurysm [78–80]. The release force of the stent-graft from the sheath of the delivery system is <30 N (Fig. 4C), which is significantly lower than our previous version (data not shown) when the stent-graft was made of a stronger metal skeleton with a union form Z-waves and the sintering process of ePTFE was not so good as it is now. The improvement is attributed to employing the integral ePTFE membrane covering and sintering technology and the low surface friction coefficient of the PTFE material.

The safety of the stent-graft and the good controllability of the new delivery system were confirmed from nine cases of large animal studies in canine models. All devices were successfully implanted in the accurate target positions and all animals survived during follow-ups. Histopathological inspections illustrated that the stent-graft had good conformability with the aorta (Fig. 6B). In addition, the smooth muscle cells and fibroblasts grew on the inner and outer surfaces of the sintered membrane, which indicated good compatibility of the ePTFE membrane with the animal aorta tissue.

The nine clinical cases strengthened the good safety and efficacy of the stent-graft and delivery system (Figs. 7 and 8). No endoleak or stent-graft failure was observed during the follow-ups, which preliminarily confirmed the good sealing performance and durability of this stent-graft. Through the analysis of the diameter of the FL measured by the follow-up CTA, all the patients got partial or complete FL thrombosis from 1 to 12 months, which indicated the efficacy of this new stent-graft in TEVAR (Fig. 8). It is worth noting that the reduction of the diameter of the proximal FL is greater than that of the middle part and distal part. This may be due to the greater blood flow entry from the proximal entry point of the AD. Once the stent-graft occludes the proximal entry point of the FL, the blood pressure in the proximal part of the FL drops rapidly. Thus, it is beneficial for the reduction of the diameter of the FL in the proximal part and remodeling of the TL.

While the limited clinical results have preliminarily indicated the promising efficacy and safety of the stent-graft system, more clinical evidence needs to be collected to verify this result. The aortic arch dissection involving the branches of the arch is still a challenge for a physician to perform a full endovascular repair in the aorta. This problem may be solved by the fenestration or chimney technique with a proper stent-graft with high performance in fatigue resistance to withstand the fenestration destruction or branch stent compression and conformability to the aortic arch.

## Conclusions

We developed a trilayer ePTFE stent-graft for interventional treatment of AD. The stent-graft with sintered ePTFE membranes sandwiched a metal stent with an asymmetric Z-wave design exhibited good mechanical and biological properties. Furthermore, we designed a delivery system with a postrelease mode, which improved the accuracy of the deployment of the stent-graft. Both the animal study and clinical trial indicated the accurate deployment of stent-graft by using the novel delivery system. The follow-ups demonstrated the safety and efficacy of the trilayer ePTFE stent-graft. The stent-graft and delivery system developed here provide an advanced medical device to treat AD patients in a mini-invasive way.

## Supplementary data

Supplementary data are available at *REGBIO* online.

## Supplementary Material

rbac049_Supplementary_Data
