# Supplementary material for: Research and clinical translation of trilayer stent-graft of expanded polytetrafluoroethylene for interventional treatment of aortic dissection
Source: Regen Biomater. 2022 Jul 22;9:rbac049. doi: 10.1093/rb/rbac049 (PMC9362767; doi:10.1093/rb/rbac049)
Supplement: rbac049_Supplementary_Data [file rbac049_supplementary_data.zip › Supporting information R1.docx]

Supporting Information

**Research and clinical translation of trilayer stent-graft of expanded polytetrafluoroethylene for interventional treatment of aortic dissection**

Gang Wang^1,2^, Caiyun Gao^1^, Benhao Xiao^2^, Jie Zhang^2^, Xunyuan Jiang^1^, Qunsong Wang^1^, Jingzhen Guo^1^, Deyuan Zhang^2^, Jianxiong Liu^2^, Yuehui Xie^2, *^, Chang Shu^3,4,^ ^*^, Jiandong Ding^1,^ ^*^

^1^State Key Laboratory of Molecular Engineering of Polymers, Department of Macromolecular Science, Fudan University, Shanghai 200438, China

^2^R&D Center, Lifetech Scientific (Shenzhen) Co., Ltd., Shenzhen 518057, China

^3^Department of Vascular Surgery, the Second Xiangya Hospital of Central South University, Changsha 410011, China

^4^State Key Laboratory of Cardiovascular Diseases, Center of Vascular Surgery, Fuwai Hospital, National Center for Cardiovascular Diseases, Chinese Academy of Medical Science and Peking Union Medical College, Beijing 100037, China

* Corresponding authors. Emails: jdding1@fudan.edu.cn (J.D. Ding); changshu01@yahoo.com (C. Shu); xieyuehui@lifetechmed.com (Y.H. Xie)

The Supporting Information contains two video files and one PDF file.

The PDF file (this file) presents supplementary methods and supplementary results with 3 supplementary figures and 2 supplementary tables shown in sequence mentioned in the main manuscript.

The two supplementary videos are uploaded in another two files as follows:

**Video S1** In vitro operation to demonstrate a stent-graft deployment process.

**Video S2** DSA images of a human clinical case to percutaneously implant a stent-graft into the thoracic aorta from the femoral artery.

**Supplementary Methods**

**Preparation of the sintered ePTFE membrane**

The bi-stretching ePTFE membrane was covered on a SUS304 mold bar with a diameter of 20 mm for 10~12 layers equal to the quantity of layers of the stent-graft. And then this assembly was placed in an oven for a heat treatment, which is similar to the stent-graft fabrication. The layers of ePTFE were bonded each other to form a cylindrical ePTFE-graft. Then the ePTFE-graft was flattened into a double layer sheet for mechanical tests or cutting into specific shapes for other chemical or biological tests.

**Thrombus formation testing**

The bi-stretching ePTFE membrane covered on a SUS304 mold bar with a diameter of 1.5 mm, and then experienced the same heat treatment process as in the stent-graft fabrication. The samples were sterilized with ethylene oxide. The thrombus formation was evaluated in a canine model. Briefly, the ePTFE covered bar was implanted into the jugular vein of a canine model for 4 hours. Limited necropsy was performed to take out the segment of vein implanted with the test sample. The samples were washed slightly by sodium chloride injection and fixed by 2.5% glutaraldehyde, dehydrated by gradient ethanol and dried. After sprayed with gold, the test samples were checked under SEM for thrombosis.

**Criteria in animal studies and clinical trials**

We used the operator grading system to evaluate the delivery system and stent-graft performance in animal studies and clinical trials. The performance indexes were specified into 5 grades (**Ⅰ-Ⅴ).**

**
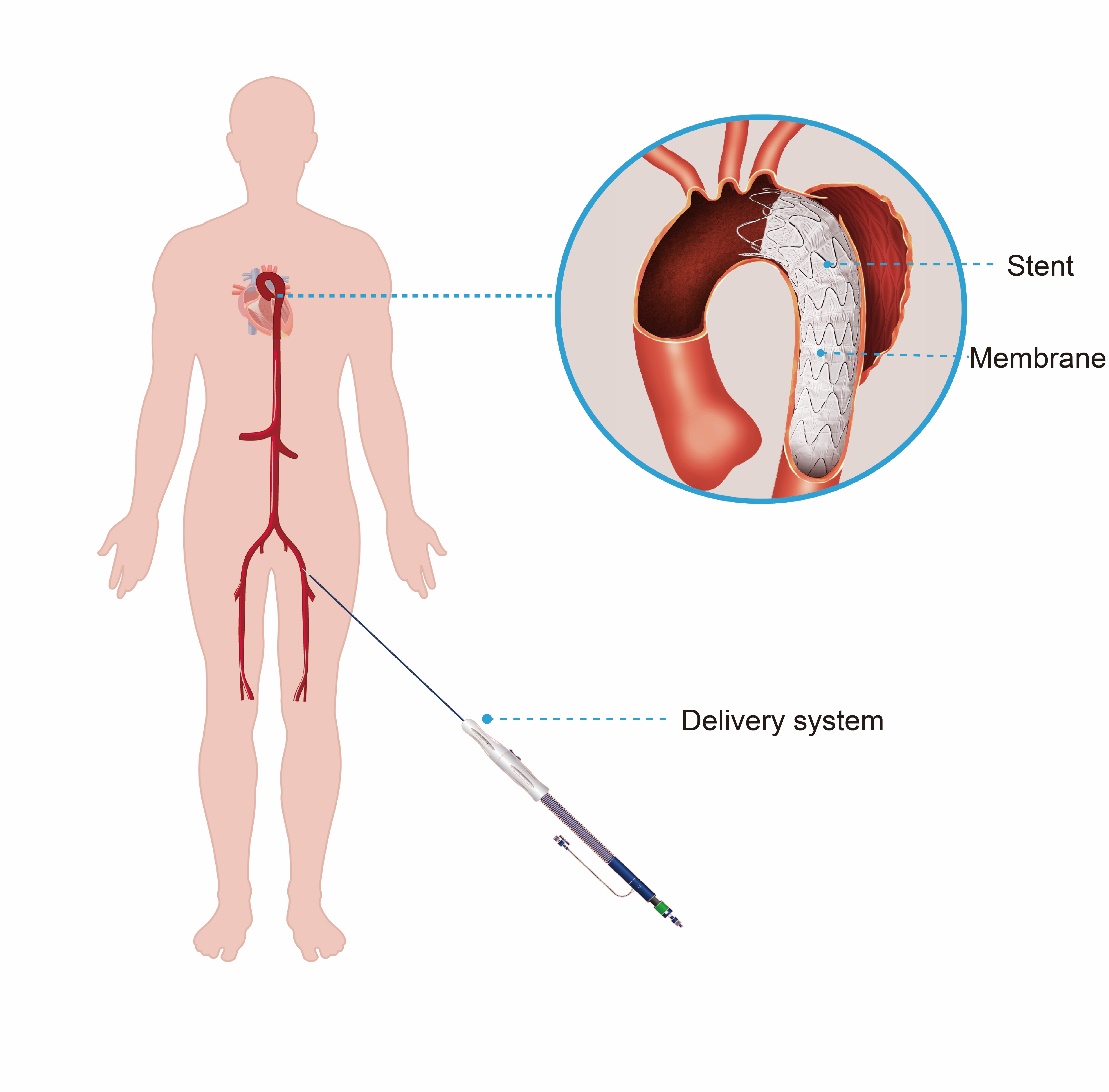
**

**Figure S1.** **Schematic presentation of the interventional procedure of a stent-graft.**

**Table S1.** **Evaluation methods of a stent-graft and its delivery system & evaluation criteria in animal studies and clinical trials.**

| **Performance index** | **Evaluation result-grade** | | | | |
| --- | --- | --- | --- | --- | --- |
|  | **Ⅰ** | **Ⅱ** | **Ⅲ** | **Ⅳ** | **Ⅴ** |
| Positioning accuracy of delivery system | Very Poor | Poor | Ordinary | Good | Excellent |
| Operational controllability of delivery system | Very Poor | Poor | Ordinary | Good | Excellent |
| Stability of delivery system | Very Poor | Poor | Ordinary | Good | Excellent |
| Supporting performance of stent-graft | Very Poor | Poor | Ordinary | Good | Excellent |
| Conformability of stent-graft | Very Poor | Poor | Ordinary | Good | Excellent |
| Attachment ability of stent-graft* | Very Poor | Poor | Ordinary | Good | Excellent |
| Visibility of stent-graft Marker | Very Poor | Poor | Ordinary | Good | Excellent |

*The attachment ability of stent-graft refers to the ability of the stent-graft to attach to the vascular wall after implantation in the human body, and it demonstrates the ability of the stent-graft to fit with the vessel.

**Supplementary Results**

**
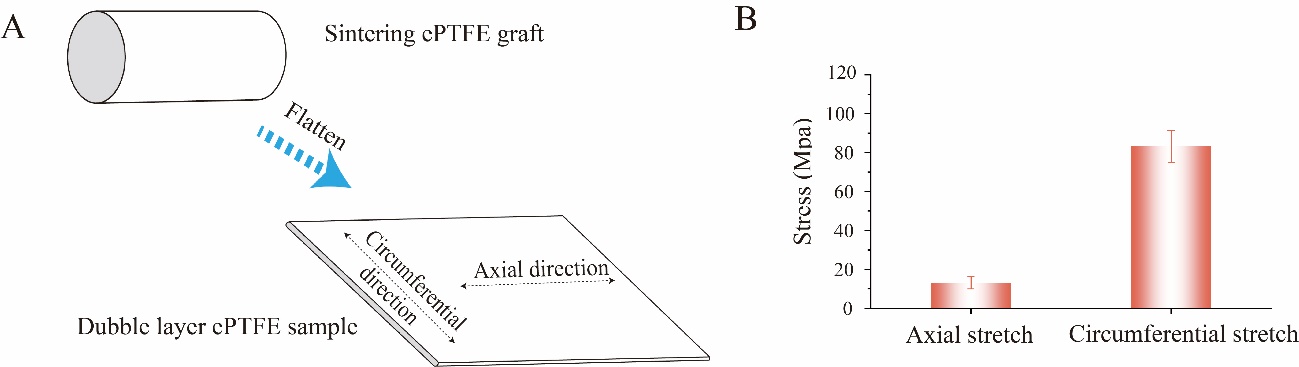
**

**Figure S2. Tensile tests to characterize mechanical properties of the sintered ePTFE graft, including axial and circumferential tensile stress.** (A) Test sample was prepared by sintering the ePTFE membranes on a cylinder mold and then flattening this cylinder graft to form a flat double layer sheet. (B) Measured tensile stresses of the graft in both axial and circumferential directions. The circumferential strength is much higher than the axial strength, which is beneficial for the artificial graft to resist the pressure of intravascular blood flow.

**
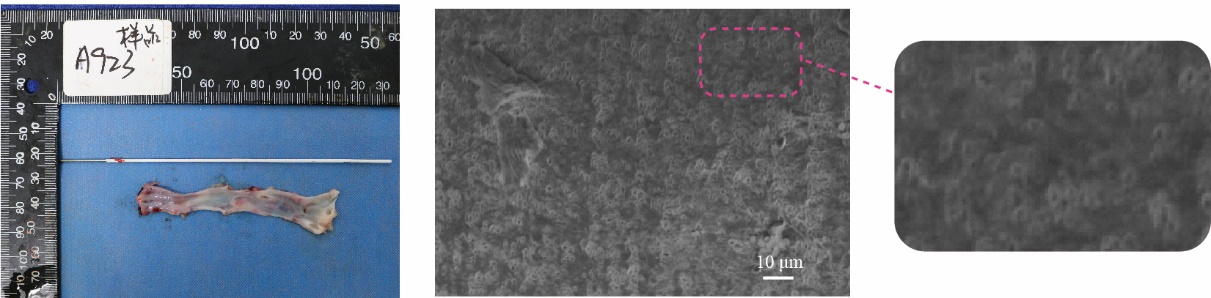
**

**Figure S3.** **Thrombus formation on the ePTFE membrane.** The left is the global view of a vessel sample and an ePTFE specimen implanted in a dog jugular vein for 4 hours, and the right is scanning electron microscopy (SEM) images of a sintered ePTFE membrane specimen taking from the animal's body. The thrombus formation test followed the protocol recommended by ISO 10993-4:2017: Biological evaluation of medical devices--Part 4: Selection of tests for interactions with blood. There was minimal to nonexistent formation of thrombus checked by the SEM image. According to ISO 10993-4: 2017, the thrombus formation score is Grade 0.

**Table S2.** **Diameter changes of aortic false lumen (FL) after stent-graft implantation compared with that of the pre-operation in 9 patients with type B aortic dissection.**

| Position | Evaluation indexes | Evaluation results (unit: mm) |
| --- | --- | --- |
| Proximal FL | Variation of diameter (1 M- Pre) | -11.4 ± 7.7 |
|  | Variation of diameter (6 M- Pre) | -13.0 ± 7.8 |
|  | Variation of diameter (12 M- Pre ) | -13.9 ± 8.0 |
| Middle FL | Variation of diameter (1 M- Pre ) | -4.3 ± 7.6 |
|  | Variation of diameter (6 M- Pre ) | -4.5 ± 7.7 |
|  | Variation of diameter (12 M- Pre ) | -6.9 ± 7.5 |
| Distal FL | Variation of diameter (1 M- Pre ) | -2.9 ± 4.1 |
|  | Variation of diameter (6 M- Pre ) | -4.4 ± 4.6 |
|  | Variation of diameter (12 M- Pre ) | -4.9 ± 5.1 |

Note: “Pre” means the pre-operation diameter.
